# Feasibility and Effectiveness of Assessing Subhealth Using a Mobile Health Management App (MibyeongBogam) in Early Middle-Aged Koreans: Randomized Controlled Trial

**DOI:** 10.2196/27455

**Published:** 2021-08-19

**Authors:** Younghwa Baek, Kyoungsik Jeong, Siwoo Lee, Hoseok Kim, Bok-Nam Seo, Hee-Jeong Jin

**Affiliations:** 1 Korean Medicine Data Division Korea Institute of Oriental Medicine Daejeon Republic of Korea

**Keywords:** mobile health, health status, mobile app, middle-aged group, subhealth, Korean medicine

## Abstract

**Background:**

Mobile health (mHealth) is a major source of health management systems. Moreover, the demand for mHealth, which is in need of change due to the COVID-19 pandemic, is increasing worldwide. Accordingly, interest in health care in everyday life and the importance of mHealth are growing.

**Objective:**

We developed the MibyeongBogam (MBBG) app that evaluates the user’s subhealth status via a smartphone and provides a health management method based on that user’s subhealth status for use in everyday life. Subhealth is defined as a state in which the capacity to recover to a healthy state is diminished, but without the presence of clinical disease. The objective of this study was to compare the awareness and status of subhealth after the use of the MBBG app between intervention and control groups, and to evaluate the app’s practicality.

**Methods:**

This study was a prospective, open-label, parallel group, randomized controlled trial. The study was conducted at two hospitals in Korea with 150 healthy people in their 30s and 40s, at a 1:1 allocation ratio. Participants visited the hospital three times as follows: preintervention, intermediate visit 6 weeks after the intervention, and final visit 12 weeks after the intervention. Key endpoints were measured at the first visit before the intervention and at 12 weeks after the intervention. The primary outcome was the awareness of subhealth, and the secondary outcomes were subhealth status, health-promoting behaviors, and motivation to engage in healthy behaviors.

**Results:**

The primary outcome, subhealth awareness, tended to slightly increase for both groups after the uncompensated intervention, but there was no significant difference in the score between the two groups (intervention group: mean 23.69, SD 0.25 vs control group: mean 23.1, SD 0.25; *P*=.09). In the case of secondary outcomes, only some variables of the subhealth status showed significant differences between the two groups after the intervention, and the intervention group showed an improvement in the total scores of subhealth (*P*=.03), sleep disturbance (*P*=.02), depression (*P*=.003), anger (*P*=.01), and anxiety symptoms (*P*=.009) compared with the control group.

**Conclusions:**

In this study, the MBBG app showed potential for improving the health, especially with regard to sleep disturbance and depression, of individuals without particular health problems. However, the effects of the app on subhealth awareness and health-promoting behaviors were not clearly evaluated. Therefore, further studies to assess improvements in health after the use of personalized health management programs provided by the MBBG app are needed. The MBBG app may be useful for members of the general public, who are not diagnosed with a disease but are unable to lead an optimal daily life due to discomfort, to seek strategies that can improve their health.

**Trial Registration:**

Clinical Research Information Service KCT0003488; https://cris.nih.go.kr/cris/search/search_result_st01.jsp?seq=14379

## Introduction

Mobile health (mHealth) using smartphone-based apps is poised to become a major source of health guidance. The “new normal” phenomenon induced by the COVID-19 pandemic is expected to further accelerate the digital economy. In health care, the representative keyword of the post-COVID-19 era is “digital (mobile) health care,” which has become a necessity. Before the COVID-19 pandemic, the main targets of health care services were existing patients and older adults. However, the COVID-19 pandemic has increased the possibility that even healthy individuals can become patients, and this has increased the demand for health care services [1-3].

The World Health Organization stated that “the use of mobile and wireless technologies to support the achievement of health objectives has the potential to transform the face of health service delivery across the globe” [4], and mHealth is already used in various areas of health care. Statista predicted that the mobile health care market would continue to grow and that the total market value for mHealth applications in the US would exceed US $50 billion in 2025, which is approximately 25 times greater than the US $2 billion value in 2016 [5]. One US survey of “app users” showed that 31% of mobile phone owners used their phones to access health information, with the largest proportion (52%) being smartphone users [6].

mHealth is being developed for the management of not only daily healthy lifestyles, including aspects such as activity level, diet [7], and smoking cessation [8], but also chronic diseases, including hypertension [9] and diabetes [10], specific diseases, including juvenile idiopathic arthritis [11] and relapsed and refractory multiple myeloma [12], and physical and emotional aspects, such as pain [13], sleep [14], and depression [15]. In recent studies by Kitt et al, mHealth was found to be effective in reducing health care costs and improving health outcomes [9,16]. It is thought that mHealth contributes to continuous and active monitoring of health at individual or group levels [6], reduces and prevents health problems through promotion of health behaviors, supports self-management of chronic diseases, and improves the knowledge of health information, which can lead to fewer visits to medical institutions and a direct reduction of medical costs [6,17,18].

Traditional East Asian medicine (TEAM), which is mainly used in China, Korea, and Japan, was included in the “Supplementary Chapter Traditional Medicine Conditions—Module I” of the 11th revision of the International Statistical Classification of Diseases and Related Health Problems (ICD-11) in 2019. This means that TEAM is now officially recognized as a part of mainstream medical practice [19]. TEAM emphasizes preventative health management before the onset of diseases and focuses on subhealth management between disease and health. The term may differ in different countries; it is termed subhealth or *mibyeong* in traditional Korean medicine (TKM) [20]. *Mibyeong* is defined as a “state of discomfort in daily life due to abnormal symptoms, or abnormal examination findings, without a diagnosis of any disease, and as a result, a decrease in capacity to recover to a healthy state” [21]. The abnormal symptoms in *mibyeong* include fatigue, pain, sleep disturbance, and digestive disturbance, as well as emotional symptoms including depression, anger, and anxiety, which are the most common reasons for people to visit clinics or health care centers [21]. Nonetheless, conventional or physiological pathology does not clearly explain why some people have a *mibyeong* status, which may carry with it a high risk for future disease development [21]. Therefore, individuals with *mibyeong* must be aware of their health status and prioritize actively managing their own health. In this study, we developed a mobile app called MibyeongBogam (MBBG), which can be accessed on a smartphone to recognize and evaluate individual subhealth status and provide individualized health management strategies based on Korean medicine [22].

The objective of this study was to assess and compare the awareness of subhealth, changes in the subjective health status, and health behaviors between intervention (MBBG use group) and control groups. Based on these results, the feasibility of the MBBG app in managing and preventing a subhealth status in individuals was assessed.

## Methods

### Study Design

**Figure 1:**
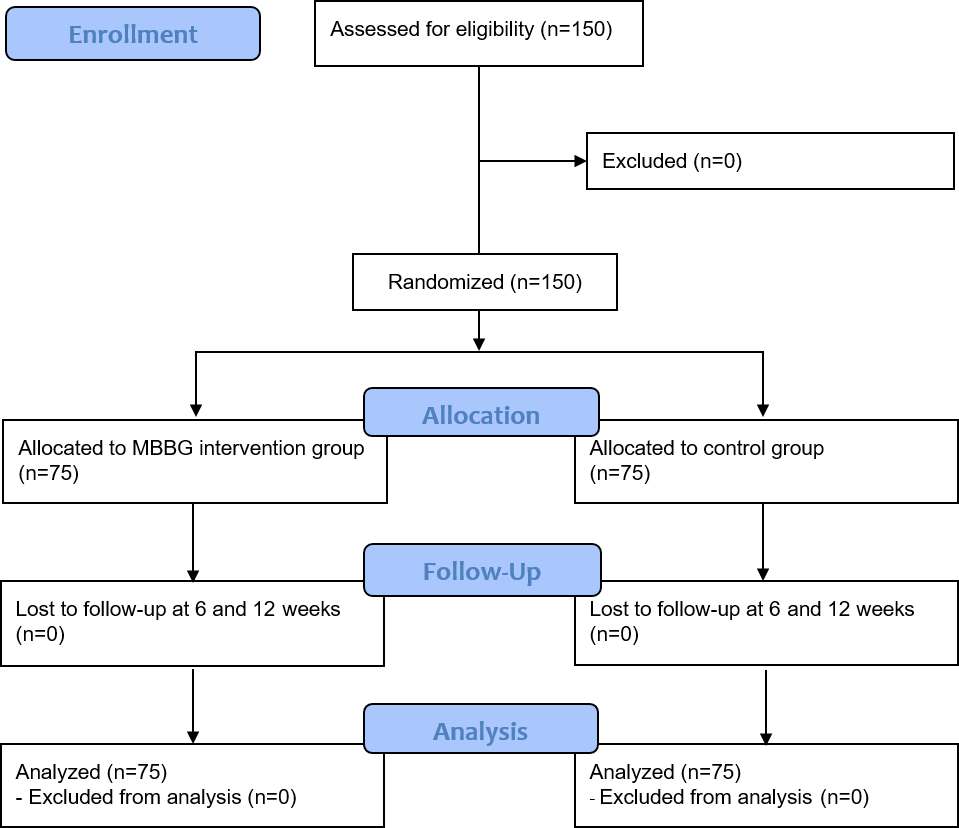
CONSORT diagram of the study. MBBG: MibyeongBogam.

This study was a prospective, open-label, parallel group, randomized controlled trial. The protocol of this study has been described in detail in a previous study [23]. Selected participants visited the hospital three times, including before the intervention, at the 6-week posttest follow-up (first follow-up), and at the 12-week posttest follow-up (second follow-up, end of the intervention), and the main outcome variables were measured at the first visit before the intervention and at 12 weeks after the intervention (Figure 1). This study was conducted from November 2018 to February 2019 in two hospitals (Kyung Hee University Korean Medicine Hospital in Hoegidont, Seoul, and Kyung Hee University Korean Medicine Hospital in Gangdong, Seoul) on a total of 150 healthy participants in their 30s and 40s without any particular health problems. The eligible participants were randomly allocated to either the MBBG or control group, at a 1:1 allocation ratio. The MBBG group used the app for a total of 12 weeks, while the control group received no intervention.

This study was approved by the Institutional Review Board at each institution (IRB numbers KOMCIRB-2018-07-002 and KHNMCOH 2018-07-002-001), and the physicians obtained written consent after all information regarding the study was provided to the participants. The protocol was registered in the Clinical Research Information Service (CRIS number KCT0003488). The anonymity and privacy of the participants were ensured as follows. Information regarding the collection and management of personal data (ie, phone number, email address, password, nickname, IP address, cookie content, etc) was provided, and consent was obtained when participants registered for the MBBG app in accordance with the Personal Information Protection Act. Moreover, app passwords were encrypted and stored in a database, and technical and physical protection measures against personal information leakage were established. Participants were also provided with personal IDs for the purpose of the study, to ensure anonymity.

### Participants

Participants were recruited via posts on both online and offline boards and were screened. Healthy male and female adults, aged between 30 and 49 years, who were capable of using mobile smartphones, were eligible for this study. They were also required to complete self-report questionnaires and undergo physical examinations. If the participants did not own mobile smartphones with Android version 4.4 or higher or iOS version 9 or higher, they were excluded from the screening process. Any participants assessed and found to have clinically significant medical conditions through an interview with a physician, from their medical history (23 disease categories)/concomitant medication reviews and physical examinations, were also excluded from the study. If they were already using other mobile health care apps, they were ineligible. Participants who were involved in other trials in the preceding month of the study or were pregnant at baseline were also deemed ineligible.

### Intervention

#### MBBG App

The intervention of this study was MBBG, a mobile app for subhealth management, developed by the Korea Institute of Oriental Medicine, Daejeon, Republic of Korea. MBBG aims to assess a user’s subhealth status, as well as their TKM-based health status, based on which it recommends specific health-promoting strategies, such as meditation, exercise, and consumption of herbal tea. Individuals can check their subhealth status and TKM health information after submitting all of the necessary information, including questionnaire responses. The questionnaires are included within the app so the participants can successfully complete these via the app. The physical examination results (height, weight, vital signs, pulse diagnosis, heart rate variability, etc) have to be inserted into the app manually or by automatic linkage [22] (Multimedia Appendix 1). In this study, all participants completed a survey questionnaire and underwent a physical examination on all three visits; only the MBBG group could access their results from the survey and physical examination by connecting with the MBBG app. The results of the control group, on the other hand, were uploaded to the MBBG app after the completion of the study.

#### MBBG App for the Intervention Group

After being allocated to the intervention group, participants first installed the MBBG app, after which they were educated on how to use the app verbally and with a user manual during each of their three visits. They were expected to use MBBG at least once daily for a total of 12 weeks. They accessed the app daily to read about their health status and ways to manage their health. In addition to hospital visits, the participants were free to complete the surveys and recommended health management protocols on the MBBG app, although this was not mandatory. The push notification function was activated to motivate and remind the participants to use the app throughout the study period. History tracking and user ranking services were also available to help promote the use of MBBG. Participants were not allowed to use any other mobile app for health management during the study period.

#### No Intervention in the Control Group

Participants allocated to the control group did not receive any intervention. They were told to maintain their usual lifestyle during the study period and were not allowed to use any mobile app for health management.

### Outcome Assessment

#### Primary Outcome

The primary outcome was the awareness of subhealth, also known as *mibyeong* in TKM. The participants were given a questionnaire consisting of the following four items: (1) “Do you know or have you heard about subhealth status?” (2) “Do you think that preventing diseases is as important as treating them?” (3) “Do you think a professional medical service aimed at managing subhealth status is necessary?” and (4) “Are you willing to use a professional medical service to manage subhealth status, if available?” Each item was then scored from 1 (not at all) to 7 (absolutely), with the total score ranging from 4 to 28. All participants were required to submit the subhealth awareness questionnaire on their first and third visits. This questionnaire was independently developed in this study and was categorized into two factors (factor 1: item 1; factor 2: items 2, 3, and 4) based on a factor analysis. The Cronbach α of the questionnaire was .52 (the Cronbach α of factor 2, excluding item 1, was .82).

#### Secondary Outcomes

Secondary outcomes included subhealth status, health-promoting behaviors, and motivation for healthy behaviors. Subhealth status was evaluated using the *Mibyeong* questionnaire, which had a satisfactory reliability (Cronbach α=.88; intraclass correlation coefficient range: 0.67-0.83 in the test-retest method) and validity (correlation range: 0.47-0.48, compared to the SF-12, which is a well-known generic health status measure) [24,25]. The *Mibyeong* questionnaire consisted of 21 items on seven symptoms (fatigue, pain, sleep disturbance, digestive disturbance, depression, anger, and anxiety), and it assessed the severity, duration, and changes in those symptoms after rest in the preceding month. Each item was evaluated on a 7-point scale, and the total score ranged from 21 (healthy) to 147 (unhealthy). Higher scores indicate poor health status. The Cronbach α in this study was .88 (the Cronbach α ranged between .78 and .92 for individual symptoms).

Health-promoting behaviors are a measure of performance of health behaviors, which were evaluated using the Health Behavior Scale [26]. This scale consists of 25 items related to health responsibility (five items), diet habits (eight items), exercise (four items), stress management (five items), and smoking habits (three items). Each item has a 4-point response, from 1 (never) to 4 (always), with the total score ranging from 25 to 100. The higher the score, the more frequently the individual engages in healthy behaviors. The Cronbach α in this study was .78 (the Cronbach α ranged between .56 and .73 for individual domains).

In addition, the motivation for engaging in healthy behaviors is a measure of confidence in health behavior practice, and it was evaluated using the Self-Efficacy Questionnaire [27,28]. The six questions therein were on people’s abilities to avoid greasy food, quit smoking, exercise regularly, take necessary medications, relieve mental stress, and obtain health-related information. The responses were provided on a 4-point scale, where 1 is “not confident at all” and 4 is “absolutely confident.” The total score ranges from 6 to 24. Higher scores indicate higher confidence in behavior practice.

### Feasibility Assessment

The feasibility of MBBG was assessed by evaluating the user finding access rate and the number of times participants logged onto the app during the intervention period. The user finding access rate was calculated using the number of times the app was accessed by the participants more than once a day, and the access rate for the 12-week intervention period was calculated.

### Sample Size

The primary objective of this study was to compare the awareness levels of subhealth between the MBBG and control groups. Since there have not been any previous studies implementing the MBBG app, we conducted another clinical trial to explore the mental health benefits of a mobile app. In that trial, there was a 0.58 effect size with a 6-week test [29]. In this study, we set the intervention period as 12 weeks, and the age for study eligibility was higher; therefore, we assumed the effect size to be, conservatively, 0.5. Thus, the sample size was calculated as 60 per group (two-sided, α=.05, and power=0.8, independent *t* test) using G-power software version 3.1.3. With a 20% dropout rate expected, we enrolled 150 participants (75 in each group).

### Random Allocation Concealment and Blinding

Randomization was performed by a statistician prior to enrollment with an assignment ratio of 1:1 and a block size of 4. Information on participant group allocation was sealed in individual opaque envelopes that were consecutively numbered for allocation concealment. The investigators opened the envelopes in consecutive order and assigned the participants to either the MBBG or the control group after a screening assessment was conducted. Since this was an open-label study, the participants and investigators were not blinded. However, the outcome assessors were blinded throughout the study to minimize possible bias.

### Statistical Analysis

In the preintervention survey, the Student *t* test and chi-square test were conducted to compare continuous and categorical variables, respectively, between the intervention and control groups. We performed an intention-to-treat analysis on all outcome measures, using the MBBG app at least once, and assessed the primary outcome at least once. Every participant participated in the study until the last day, and no values were excluded. An analysis of covariance (ANCOVA) was performed to compare the effects of the primary and secondary outcomes between the two groups after MBBG intervention. Age, sex, BMI, and the baseline value of each outcome variable were adjusted to calculate the least square means and standard errors. All analyses were performed using SAS 9.4 software (SAS Institute Inc), and statistical significance was set at *P*<.05.

## Results

### Study Population

A total of 150 participants were included in the study. Of these participants, 75 were randomly assigned to the MBBG group (23 men and 52 women) and 75 were assigned to the control group (21 men and 54 women). There were no differences in general characteristics, such as sex, age, and BMI, between the two groups, and the outcome variables were similar between the two groups, except for some specific variables, including total score of the subhealth status, pain, anger, and anxiety (Table 1).

**Table 1:** Participants’ baseline demographics and outcome variable characteristics.

Variable		MBBG^a^ group (n=75)	Control group (n=75)	*P* value
Sex (men/women), n		23/52	21/54	.86
Age (years), mean (SD)		41.73 (5.17)	42.09 (4.74)	.66
BMI (kg/m^2^), mean (SD)		23.66 (3.61)	24.21 (4.06)	.38
Subhealth awareness score, mean (SD)		21.13 (2.85)	21.48 (3.08)	.48
**Subhealth status (*mibyeong*) score, mean (SD)**				
	Total score	35.90 (14.20)	40.80 (12.40)	.03^b^
	Fatigue	9.01 (4.17)	9.33 (3.16)	.60
	Pain	4.77 (3.89)	6.31 (3.77)	.02^b^
	Sleep disturbance	5.17 (4.11)	5.36 (3.35)	.76
	Digestive disturbance	4.76 (3.27)	4.89 (1.96)	.76
	Depression	4.55 (3.41)	4.91 (2.02)	.43
	Anger	3.65 (2.26)	4.99 (2.27)	<.001^b^
	Anxiety	4.03 (2.86)	5.00 (2.34)	.02^b^
**Health-promoting behavior score, mean (SD)**				
	Total score	66.05 (8.40)	66.92 (0.97)	.53
	Health responsibility	15.24 (2.74)	15.20 (2.38)	.93
	Exercise	10.37 (2.50)	10.35 (2.52)	.95
	Diet habits	17.36 (3.52)	18.35 (3.22)	.08
	Stress management	13.07 (2.43)	13.00 (2.44)	.87
	Smoking habits	10.01 (2.64)	10.03 (2.82)	.98
Motivation for healthy behaviors		18.85 (2.38)	19.14 (2.47)	.46

^a^MBBG: MibyeongBogam.

^b^*P*<.05.

### Subhealth Effectiveness Assessment

Subhealth awareness, which is a primary outcome, tended to slightly increase for both groups after the MBBG intervention; however, there was no significant difference in the score between the two groups (MBBG group: mean 23.69, SD 0.25 vs control group: mean 23.1, SD 0.25; *P*=.09). For secondary outcomes, several variables of subhealth status showed significant differences between the two groups. In the MBBG group, subhealth total score, sleep disturbance, depression, anger, and anxiety improved compared to the findings in the control group (Table 2).

**Table 2:** Results of the subhealth effectiveness assessment using primary and secondary outcome measures at the 12-week follow-up after the intervention.

Variables			MBBG^a^ group (n=75), least square mean (SE)		Control group (n=75), least square mean (SE)		*F* ^b^		*P* value^b^
Subhealth awareness			23.69 (0.25)		23.1 (0.25)		2.94		.09
**Subhealth status (mibyeong) score**									
	Total score	33.94 (1.10)		37.5 (1.10)		5.13		.03^c^	
	Fatigue	8.56 (0.37)		7.95 (0.37)		1.35		.25	
	Pain	5.02 (0.36)		5.57 (0.36)		1.12		.29	
	Sleep disturbance	4.52 (0.33)		5.57 (0.33)		5.18		.02^c^	
	Digestive disturbance	4.47 (0.25)		5.05 (0.25)		2.69		.10	
	Depression	3.85 (0.23)		4.85 (0.23)		9.07		.003^c^	
	Anger	3.51 (0.22)		4.36 (0.22)		6.79		.01^c^	
	Anxiety	3.68 (0.22)		4.51 (0.22)		6.93		.009^c^	
**Health-promoting behaviors**									
	Total score	68.27 (0.67)		68.47 (0.67)		0.04		.84	
	Health responsibility	15.83 (0.24)		15.80 (0.24)		0.05		.83	
	Exercise	10.39 (0.18)		10.46 (0.18)		0.08		.78	
	Diet habits	18.47 (0.26)		18.29 (0.26)		0.24		.62	
	Stress management	13.25 (0.21)		13.43 (0.21)		0.36		.55	
	Smoking habits	10.39 (0.18)		10.47 (0.18)		0.10		.75	
Motivation for healthy behaviors			19.32 (0.20)		18.92 (0.20)		1.82		.18

^a^MBBG: MibyeongBogam app.

^b^ANCOVA analysis adjusted for sex, age, BMI, and the baseline value of each outcome variable.

^c^*P*<.05.

### Feasibility Assessment

The retention rate was assessed by evaluating the user finding access rate of the MBBG app during the intervention period, and the retention rate was 75.1% (SD 15.9%, range 22%-100%) for the entire 12-week period. In particular, the mean access rate for the first 6 weeks postintervention was 71.9% (SD 17.7%, range 25%-100%), and the mean access rate for the next 6 weeks was 78.8% (SD 16.3%, range 14%-100%).

## Discussion

### Principal Results

This study is the first to compare changes in subhealth awareness and subhealth status after 12 weeks of using the MBBG app, which was developed as a framework based on the concept and management methods of TKM. This study also assessed the feasibility of the app as a self-guided preventative intervention. First, there was no significant difference in subhealth awareness between the MBBG and control groups; however, subhealth awareness tended to slightly increase in both groups. Second, the MBBG app showed positive effects on sleep, depression, anger, and anxiety, which are related to mental health. However, health-promoting behaviors and motivation for healthy behaviors were not significantly improved. This study is meaningful in that the MBBG app had significant effects on improving the health status in healthy adults, particularly the management of mental health symptoms.

### Comparison With Prior Work

#### Awareness of Subhealth

In our study, there was no significant difference in the awareness of subhealth, which was a primary outcome, between the two groups. However, it tended to increase in both groups regardless of MBBG app usage. In our study, all participants in both groups met the researcher three times. The participants then received explanations on health and participated in health-related surveys. We suggest that processes, such as receiving explanations about the study before consenting to participate, completing health-related questionnaires at each visit, and the health examination processes of measuring blood pressure and heart rate, would have partially contributed to the increased interest in participants’ health awareness regardless of MBBG app usage. In a meta-analysis of mental health apps, using apps involving contact with medical staff was less effective than using apps without in-person feedback [30]. This is because a standalone app that does not promote contact with medical staff can enhance personal privacy and autonomy [31]. However, the main objective of our study was to assess changes in health awareness through the use of the MBBG app, that is, whether the participants became aware of the necessity of health care. Therefore, unlike the intervention effects of health apps observed in previous studies, it is thought that health-related information provided by medical staff, who were in contact with the participants, was an important factor of health awareness in our study. Additionally, previous studies that performed path analysis of cognitive factors related to the use of health apps demonstrated that the health consciousness of individual participants directly affected the use of health apps [32]. In our study, the mean pretest score of health awareness in the MBBG group was 21 out of 28, and a similar score was observed in the control group, suggesting that the participants in our study were already highly interested in health, which may be related to their health awareness.

#### Improvement of Mental Health

Interestingly, the subhealth status significantly improved in the MBBG group compared to the control group. Significant differences were observed in mental health aspects, such as sleep, depression, anger, and anxiety, between the two groups. These findings suggest that the MBBG app can improve mental health, especially discomfort, which is commonly observed in everyday life. The participants in our study belonged to the early middle-aged group, and these individuals often experience problems related to sleep, such as insufficient sleep time [33,34], decreased quality of sleep [35], and anxiety and depression symptoms [36]. Such symptoms are highly related to obesity, metabolic syndrome, and cardiovascular diseases [33,34,37,38]. However, most people do not seek or receive proper treatment for mental health problems. Recently, many scholars have predicted that technology-based interventions, such as health apps, have the potential to reduce treatment gaps in mental health. In addition, it is predicted that mental health apps will not replace the role of medical professionals in digital mental health and instead will play a role in interventions [39]. Moreover, a high level of evidence for the effects of smartphone-based interventions for common mental health problems, such as depressive symptoms, anxiety symptoms, stress levels, general psychiatric distress, quality of life, and positive effects, has been observed [40]. Approximately 41% of smartphone-based apps for mental health were developed for symptom relief, and these apps can help improve minor outcomes such as relaxation [41]. Furthermore, studies have reported that developing interest in mental health, acknowledging the problem, and undergoing interventions that can resolve minor symptoms at individual levels through health apps have positive effects on mental health in adults [40]. Therefore, the MBBG app developed in this study could serve as a health guide for those with physical and mental discomfort and those who cannot visit the appropriate hospital at the right time. A personalized health management strategy based on individual Korean medicine characteristics and discomfort is referred to as *Yangseng* in Korean medicine. This management strategy is further divided into herbal medicine, acupressure, exercise, and food in the MBBG app. Therefore, further studies on the positive effects of the MBBG app as an intervention in digital mental health care are required.

#### Change in Health Behaviors and Motivation

Health-promoting behaviors and motivation for health behaviors were not significantly different between the MBBG and control groups. Items on health responsibility (consultations with medical staff, health-related information acquisition, regular health examinations, etc), exercise (walking, high intensity exercise, etc), diet habits (regular meals, balanced food intake, etc), stress management (comfortable mindset, comfortable mindset, etc), and smoking habits (smoking cessation, overcoming the urge to smoke, etc) were used to assess the practice of and confidence in health-promoting behaviors. However, health-promoting behaviors and motivation did not significantly improve with MBBG app usage. A study by Ernsting et al focused on the use of health apps related to health-promoting behaviors such as smoking cessation, healthy diet, and weight loss. However, the authors argued that using health apps does not necessarily reflect the practice of health behaviors, but rather the motivation of users to change their health behaviors [42]. In addition, two systematic literature review studies reported different findings on the association between health apps and health-promoting behaviors. In the literature review of Lee et al on 12 studies that used health apps for health promotion programs, mobile app programs for the general public were mostly used for weight management and improvement of physical activities, and the effects of health-promoting behaviors were observed in those who used the apps for specific purposes compared to those who did not use the apps [43]. In contrast, in a study that reviewed 52 randomized controlled trials published between 2014 and 2019, there was no strong evidence to support the effects of mobile apps on improving health behaviors or outcomes [7]. Likewise, in this study, there was no significant difference between the MBBG and control groups. Therefore, it would be necessary to conduct a follow-up study by selecting appropriate participants and employing a detailed study design to assess the health-promoting effects of the MBBG app.

Lastly, the mean retention rate of the MBBG app in this study was 75.1%, which is similar to the rate of 79.6% (minimum 29%, maximum 100%) observed in a previous study [44]. In addition, the retention rate was defined as the number of initial study participants who remained in the study through the intervention period and follow-up in previous studies. In our study, the retention rate also included the daily app access rate of the participants, which reflected a high compliance. Similar results were observed in the dropout rate of participants. Although a dropout rate of 20% was predicted when designing the study and calculating the number of participants to include, the actual dropout rate was 0%. First, the participants of this study were between the ages of 30 and 40 years and were comfortable or familiar with using mobile apps. A previous study reported that 44.3% of those aged between 30 and 40 years used health apps, which is higher than the proportion of app users in other age groups [45]. Second, this study was a feasibility study that assessed the use of the MBBG app and the change in awareness of subhealth. Thus, it is likely that the high degree of autonomy provided to the participants contributed to the low dropout rate.

### Limitations

This study has several limitations. First, this study was conducted on participants in the early middle-age group. Therefore, generalization of the results to other age groups would be limited. However, this study is clinically and academically meaningful in that the feasibility of the app was evaluated in individuals in their 30s and 40s who required or needed to start taking more interest in health care. Second, the purpose of this study was to assess health status awareness and the feasibility of the MBBG app. Therefore, we could not assess whether the health management methods suggested by the MBBG app were implemented by the participants. Future studies should focus on the management strategies provided by the MBBG app and assess its effects. Third, only 150 participants were included in the study, and the 12-week intervention period was not long enough. However, the sample size in our study was similar to or slightly larger than that in other studies on mHealth interventions [29,30], and the intervention period was also similar to that in previous studies, which was 4-24 weeks [30]. Lastly, the participants and researchers were not blinded to randomization, which could have caused biased results. However, randomization was performed to control for adjusted variables, such as sex and age, which mainly affected the outcome variables between the two groups.

### Conclusions

This randomized controlled trial compared the perception of and changes in the health status between intervention and control groups by using the MBBG app as an intervention for 3 months, and examined the possibility of using the MBBG app as a self-guided preventative intervention.

The MBBG app was developed to provide personalized health management strategies based on individual characteristics and self-awareness of the health status, which was assessed using symptoms, such as fatigue, sleep, and depression, which are commonly observed in daily life. In this study, the MBBG app did not significantly improve subhealth awareness. However, the MBBG app showed potential for improving health outcomes, especially in the mental health aspect, of individuals without particular health problems. We believe that the MBBG app would be useful for members of the general public, who are not diagnosed with a disease but do not enjoy optimal daily life due to discomfort, to seek strategies that can improve their health. Based on the feasibility of the app observed in this study, a large-scale randomized controlled trial would be necessary in the future. Detailed health status (eg, symptom types such as sleep disturbance and depression), specific health-promoting behaviors, and strategies to stimulate motivation based on user convenience are needed to evaluate the effects of the MBBG app. However, expansion of the contents of the MBBG app and development of customized health care guidelines should be prioritized before conducting a large-scale study.

## Appendix

Multimedia Appendix 1 MibyeongBogam app content.

Multimedia Appendix 2 CONSORT-eHEALTH checklist (V 1.6.1).

## Abbreviations

MBBG: MibyeongBogam
mHealth: mobile health
TEAM: traditional East Asian medicine
TKM: traditional Korean medicine
