# Supplementary material for: Feasibility and Effectiveness of Assessing Subhealth Using a Mobile Health Management App (MibyeongBogam) in Early Middle-Aged Koreans: Randomized Controlled Trial
Source: JMIR Mhealth Uhealth. 2021 Aug 19;9(8):e27455. doi: 10.2196/27455 (PMC8414299; doi:10.2196/27455)
Supplement: Multimedia Appendix 1 [file mhealth_v9i8e27455_app1.docx]

**Multimedia Appendix 1.** MibyeongBogam app content.

| **Contents** | **Description** | **Contents** | **Description** |
| --- | --- | --- | --- |
| **(A) Application screen** | | **(B) Main screen** | |
| 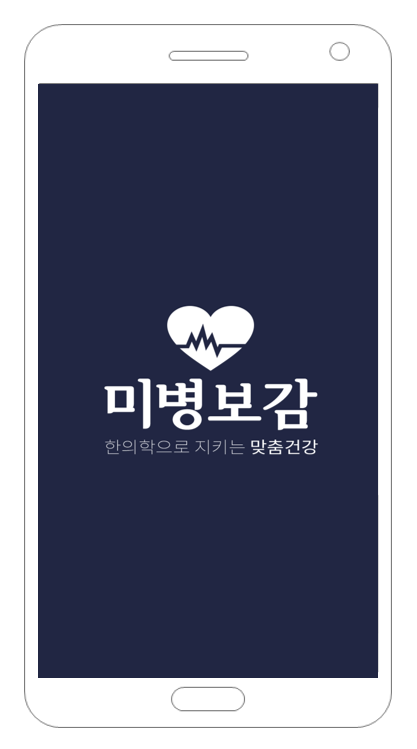 | MibyeongBogam  Personalized health care with Korean medicine | 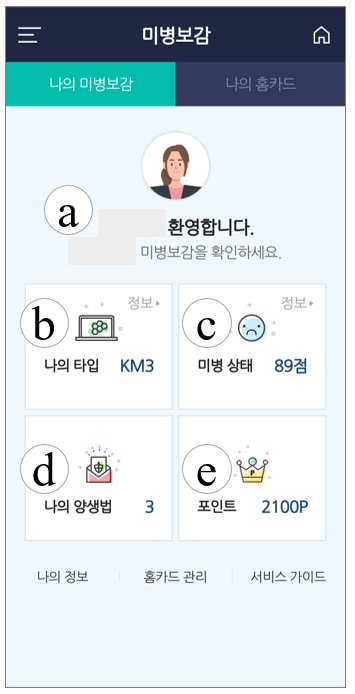 | a. Welcome [name]. Check your MibyeongBogam!  b. My type  c. My Subhealth status  d. My Yangseng method  e. Point |
| **(C) Assessment of health status Ⅰ** | | **(D) Assessment of health status Ⅱ** | |
| 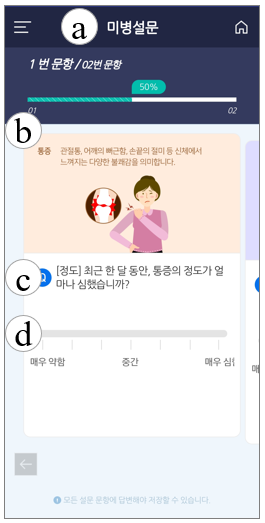 | a. Subhealth status: Mibyeong Questionnaire  b. Pain: various discomfort around the body including joints, shoulder, and fingers.  c. Question: [severity] How severe was the pain in the last month?  d. Answer: Very weak – Moderate –Very severe | 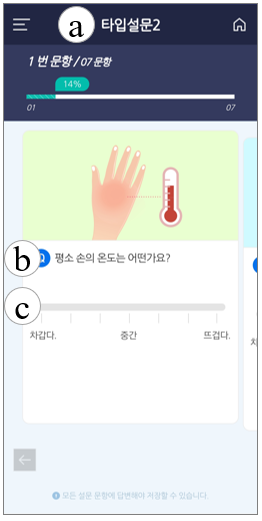 | a. Type Questionnaire 2  b. Question: How is the normal temperature of your hand?  c. Answer: Cold – Neutral – Hot (Warm) |
| **(E) My subhealth status** | | **(F) Analysis of health status** | |
| 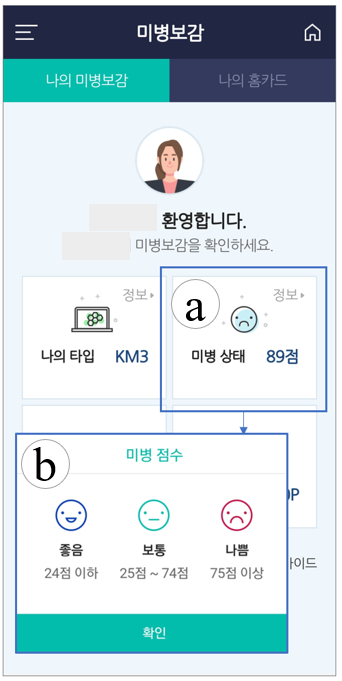 | a. My Subhealth status (score)  b. Criteria of Subhealth status  heathy (≤ 24), moderate (25 – 74), unhealthy (≥ 75) | 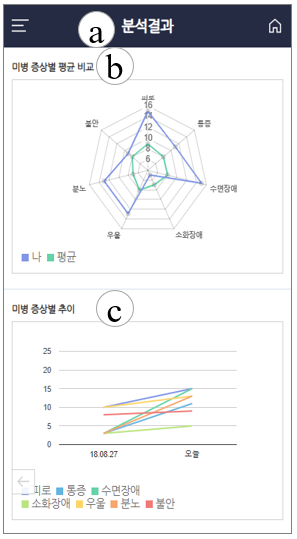 | a. Result of Subhealth status  b. Comparison of mean scores of subhealth status  (Top to right) Fatigue, pain, sleep disturbance, digestive disturbance, depression, anger, anxiety  c. Trend of each symptom |
| **(G) Health management methods** | | **(H) Health management methods (Recommendation)** | |
| 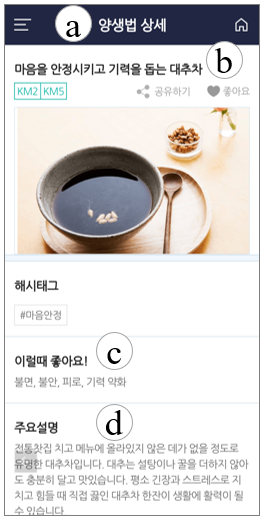 | a. Details of the Yangseng method  b. Jujube tea, relaxes your mind, and restores energy  c. It's good for!  Sleep disturbance, anxiety, fatigue, and decreased energy  d. Main explanation  Jujube tea is a famous tea that is served in almost every traditional tea house. Jujube is sweet and savory even without any sugar or honey. It can energize your life when you are tired and stressed. | 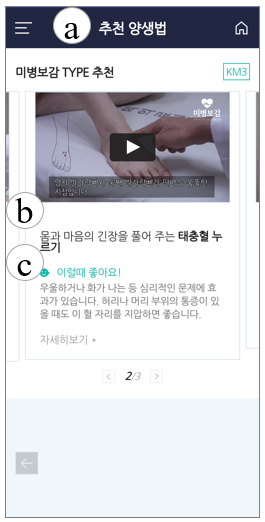 | a. Recommended Yangseng method  b. Press on LR3 to relieve tension in the body and mind  c. It's good for!  Psychological problems such as depression and anger. It is also good to press on LR3 when you have headache or pain in the back. |
